# Nebulized C1-Esterase Inhibitor does not Reduce Pulmonary Complement Activation in Rats with Severe *Streptococcus Pneumoniae* Pneumonia

**DOI:** 10.1007/s12013-016-0766-1

**Published:** 2016-09-28

**Authors:** Friso de Beer, Wim Lagrand, Gerie J. Glas, Charlotte J. P. Beurskens, Gerard van Mierlo, Diana Wouters, Sacha Zeerleder, Joris J. T. H. Roelofs, Nicole P. Juffermans, Janneke Horn, Marcus J. Schultz

**Affiliations:** 1 Laboratory of Experimental Intensive Care and Anesthesiology (L·E·I·C·A), Academic Medical Center, University of Amsterdam, Amsterdam, The Netherlands; 2 Department of Intensive Care, Academic Medical Center, University of Amsterdam, Amsterdam, The Netherlands; 3 Department of Immunopathology, Academic Medical Center, University of Amsterdam, Amsterdam, The Netherlands; 4 Department of Hematology, Sanquin Research and Landsteiner laboratory, Academic Medical Center, Amsterdam, The Netherlands; 5 Department of Pathology, Academic Medical Center, University of Amsterdam, Amsterdam, The Netherlands

**Keywords:** Pneumonia, Complement, Complement activation, Pulmonary complement activation, Lung injury, Pulmonary inflammation, Lung inflammation, C1-esterase inhibitor, Complement inhibition

## Abstract

Complement activation plays an important role in the pathogenesis of pneumonia. We hypothesized that inhibition of the complement system in the lungs by repeated treatment with nebulized plasma-derived human C1-esterase inhibitor reduces pulmonary complement activation and subsequently attenuates lung injury and lung inflammation. This was investigated in a rat model of severe *Streptococcus pneumoniae* pneumonia. Rats were intra–tracheally challenged with *S. pneumoniae* to induce pneumonia. Nebulized C1-esterase inhibitor or saline (control animals) was repeatedly administered to rats, 30 min before induction of pneumonia and every 6 h thereafter. Rats were sacrificed 20 or 40 h after inoculation with bacteria. Brochoalveolar lavage fluid and lung tissue were obtained for measuring levels of complement activation (C4b/c), lung injury and inflammation. Induction of pneumonia was associated with pulmonary complement activation (C4b/c at 20 h 1.24 % [0.56–2.59] and at 40 h 2.08 % [0.98–5.12], compared to 0.50 % [0.07–0.59] and 0.03 % [0.03–0.03] in the healthy control animals). The functional fraction of C1-INH was detectable in BALF, but no effect was found on pulmonary complement activation (C4b/c at 20 h 0.73 % [0.16–1.93] and at 40 h 2.38 % [0.54–4.19]). Twenty hours after inoculation, nebulized C1-esterase inhibitor treatment reduced total histology score, but this effect was no longer seen at 40 h. Nebulized C1-esterase inhibitor did not affect other markers of lung injury or lung inflammation. In this negative experimental animal study, severe *S. pneumoniae* pneumonia in rats is associated with pulmonary complement activation. Repeated treatment with nebulized C1-esterase inhibitor, although successfully delivered to the lungs, does not affect pulmonary complement activation, lung inflammation or lung injury.

## Introduction

Severe pneumonia is associated with high morbidity and mortality [1]. While early and adequate antimicrobial therapy remains the cornerstone of treatment, additive therapeutic strategies may further improve outcome. The complement cascade plays an important role in pulmonary host defense against invading pathogens [2]. While complement protein deficiencies are associated with severe and recurrent pulmonary infections [3, 4], excessive complement activation has been found to be associated with development of lung injury [5–7]. Thus, a balanced regulation of the local complement system seems to be important to maintain homeostasis during severe pneumonia.

The complement cascade is under control of various proteins. C1-esterase inhibitor (C1-INH) is the major negative regulator of the classical and lectin complement pathway [8]. During acute inflammation, a relative C1-INH deficiency may occur [9]. This could result from inactivation due to elastase released from host neutrophils combined with invading pathogens resulting in overwhelming stimuli [10, 11]. C1-INH has been used as treatment in various disease models [12–18], such as myocardial infarction and septic shock, showing beneficial results on myocardial infarction size and improved renal function.

C1-INH treatment is scarcely investigated in models of lung injury. Despite a reduction in pulmonary complement activation in one experiment, lung inflammation and lung injury were not affected [19, 20]. In these experiments C1-INH was administered systemically, possibly resulting in too low local levels to have beneficial effects. We hypothesized that locally applied C1-INH, by repeated nebulization, inhibits pulmonary complement activation and subsequently mitigates lung injury. This was investigated in a placebo-controlled experimental study in a well-established rat model of severe *Streptococcus pneumoniae* pneumonia.

## Methods

### Animals

**Fig. 1:**
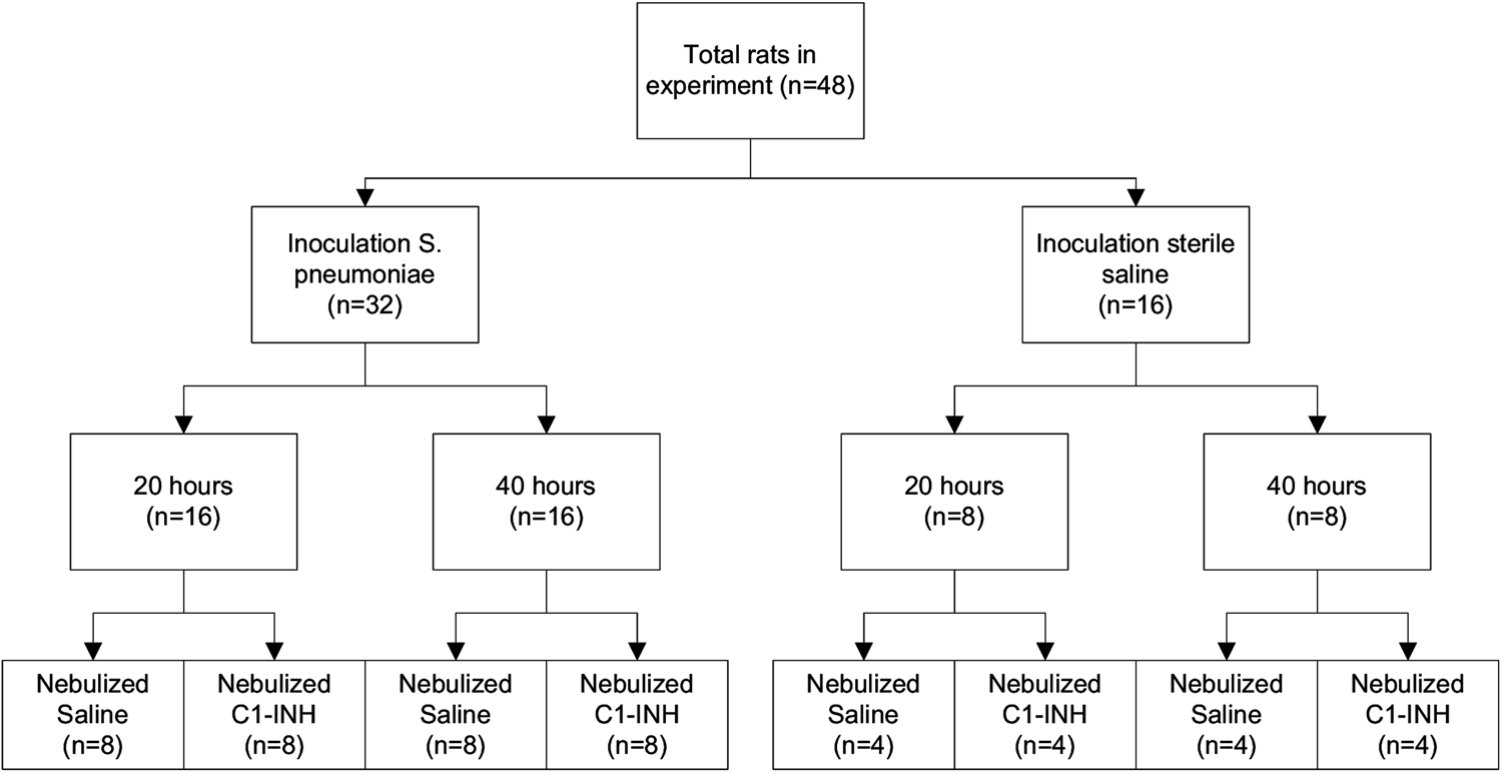
Consort diagram of the study

Our institutional animal care and use committee approved the study protocol. Procedures were followed through in agreement with the institutional Standards for Human Care and Use of Laboratory Animals. A flow diagram of the study is presented in Fig. 1.

### Induction of Pneumonia

Thirty-two male Sprague–Dawley rats (Harlan, The Hague, The Netherlands) weighing 250–300 g were challenged intra–tracheally with 250 µL containing~1.0 × 10^7^ colony forming units (CFU) of *S. pneumoniae* serotype 3 (American Type Culture Collection 6303, Rockville, MD, USA). Administration of this solution was performed under mild anesthesia (3 % isoflurane in oxygen) using a trans–oral miniature nebulizer (Penn–Century, Philadelphia, PA, USA). Sixteen rats received sterile saline (0.9 % NaCl) and served as controls.

### Treatment with C1-INH

Rats were randomized to receive treatment with either 200 IU of C1-INH in a volume of 2 ml (Sanquin, Amsterdam, The Netherlands) or an equal volume of placebo (0.9 % NaCl) using an Aeroneb Pro nebulizer (Aerogen Ltd., Galway, Ireland). Rats with pneumonia and healthy controls were repeatedly exposed to nebulized C1-INH or placebo, as described previously [21], using an exposure system in which the noses of the rats are directly exposed to the nebulized C1-INH or saline. The nebulizing system consists of a circular central chamber distributing the aerosols to the connected restraint tubes (CHT 249 restraint tube, CH technologies Inc., Westwood, NJ, USA). The system allows for nebulization of several rats simultaneously. Nebulization was performed 30 min before induction of pneumonia and every 6 h thereafter. Rats were sacrificed 20 or 40 h after inoculation to investigate C1-INH treatment at two time points.

### Primary and Secondary Outcomes

For our primary outcome we measured activated complement component 4 (C4b/c) in bronchoalveolar lavage fluid (BALF) as marker of classical/lectin pathway activation in the pulmonary compartment. Secondary endpoints were lung injury and lung inflammation. Lung injury was determined measuring total histopathology score, relative lung wet weight, total protein, and neutrophil influx. A pro-inflammatory cytokine profile of the lung provided the level of pulmonary inflammation (tumor necrosis factor (TNF)-α, interleukin (IL)-6 and cytokine-induced neutrophil chemoattractant (CINC)-3).

### Harvesting of Blood, BALF, and Lung Tissue

After 20 or 40 h, rats with *S. pneumoniae* pneumonia and controls were anesthetized by intraperitoneally injecting a solution containing 90 mg/kg ketamine (Nimatek, Eurovet Animal Health BV, Bladel, The Netherlands), 0.125 mg/kg dexmedetomidine (Dexdomitor, Janssen Pharmaceutica NV, Beerse, The Netherlands) and 0.05 mg/kg atropine (Atropinesulfate, Centrafarm BV, Etten–Leur, The Netherlands). Thereafter, rats were exsanguinated by puncture of the inferior vena cava. Blood was collected in a 4 mL Ethylenediaminetetraacetic acid (EDTA) tube (Vacutainer, Becton Dickinson B.V., Breda, The Netherlands) and thereafter centrifuged for 10 min at 1800 × g at 4 °C. The lungs were removed in total and after ligation of the right lung, the left lung was lavaged three times with 2 mL of sterile saline containing 10 % EDTA. After removal, the top of the right lung was used for histopathology and was fixed in 4 % buffered formaldehyde and subsequently embedded in paraffin. One part of the right lung was used for relative lung wet weight determination and the rest was homogenized for CFU counts. Cell counts in BALF were performed using a hematocytometer (Z2 Coulter Particle Counter; Beckman Coulter Corporation, USA) and Giemsa–stained cytospin preparations (Dade Behring AG, Dudingen, Switzerland) were used for differential counts. Hereafter, BALF was centrifuged for 10 min at 300 × g at 4 °C.

### Bacterial Outgrowth in Lung and Blood

CFU counts in lung homogenates were determined by culturing 10-fold dilutions on blood agar plates. Dissemination of bacteria was determined by culturing 100 μL of blood directly obtained from the vena cava inferior on blood agar plates (AMC, Amsterdam, The Netherlands). Blood agar plates were incubated at 37 °C in 5 % CO_2_ and CFUs were counted the following day.

### Lung Homogenization

To determine pulmonary inflammation in lung homogenate, lungs were weighed and homogenized in 4 times its weight of sterile normal saline (i.e., 4 × lung weight [mg] in μL) using a tissue homogenizer (Biospec Products, Bartlesville, OK, USA). Lung homogenates were then diluted 1:1 in lysis buffer (105 nmol/L NaCl; 15 mmol/L Tris; 1 mmol/L MgCl_2_–H_2_O; 1 mmol/L CaCl_2_; 1 % Triton X-100; and 100 μg/mL of a mixture of protease inhibitors; pepstatin A, leupeptin, and aprotinin) and after centrifuging a cell and debris free supernatant was obtained.

### Assays

Human C1-INH antigen and activity, as well as activated complement factor 4 were measured using an ELISA (Sanquin, Amsterdam, The Netherlands) as described before [22]. The assay for activated complement factor 4 is referred to as C4b/c since no distinction is made between C4b, C4bi and C4c. Levels of C4b/c are depicted in percentage (%) of maximal level of activation in plasma. Levels of TNF-α, IL-6, and CINC-3 in lung homogenate were measured using ELISA (R&D Systems, Abingdon, UK).

### Histopathology Scores

Paraffin embedded lung tissue sections of four µm thick were stained with haematoxylin and eosin. Thereafter, tissue sections were scored by a pathologist blinded to group randomization. To score lung injury, different variables of the lung sections were quantitatively analyzed. Interstitial inflammation, endothelialitis, bronchitis, edema, pleuritis, and thrombus formation were graded on a scale of 0–4 (0, absent; 1, mild; 2, moderate; 3, severe; 4, very severe) as described previously [23]. The total histopathological lung injury score comprises the sum of scores for all variables.

### Statistical Analysis

Data of individual rats are shown in graphs with median showing variety in distribution. In text data are presented as median with range. To investigate the effect of 20 or 40 h of *S. pneumoniae* pneumonia on pulmonary complement activation, lung injury and inflammation, rats were compared to their respective healthy control. To investigate the effect of treatment with nebulized plasma-derived human C1-INH on *S. pneumoniae* pneumonia induced pulmonary complement activation, lung injury and inflammation, C1-INH treated rats were compared with placebo treated rats at 20 and 40 h, respectively. For comparisons of two groups, student’s *t*-test or Mann Whitney U was used according to data distribution. For multiple group comparisons, ANOVA with Bonferroni post-hoc test or Kruskall–Wallis test with a Dunn’s post-hoc correction was used according to the data distribution.

Statistical analysis was performed using GraphPad Prism version 5 (GraphPad software Inc, La Jolla, CA, USA). A *p*-value of <0.05 was considered statistically significant.

## Results

### Animals

**Fig. 2:**
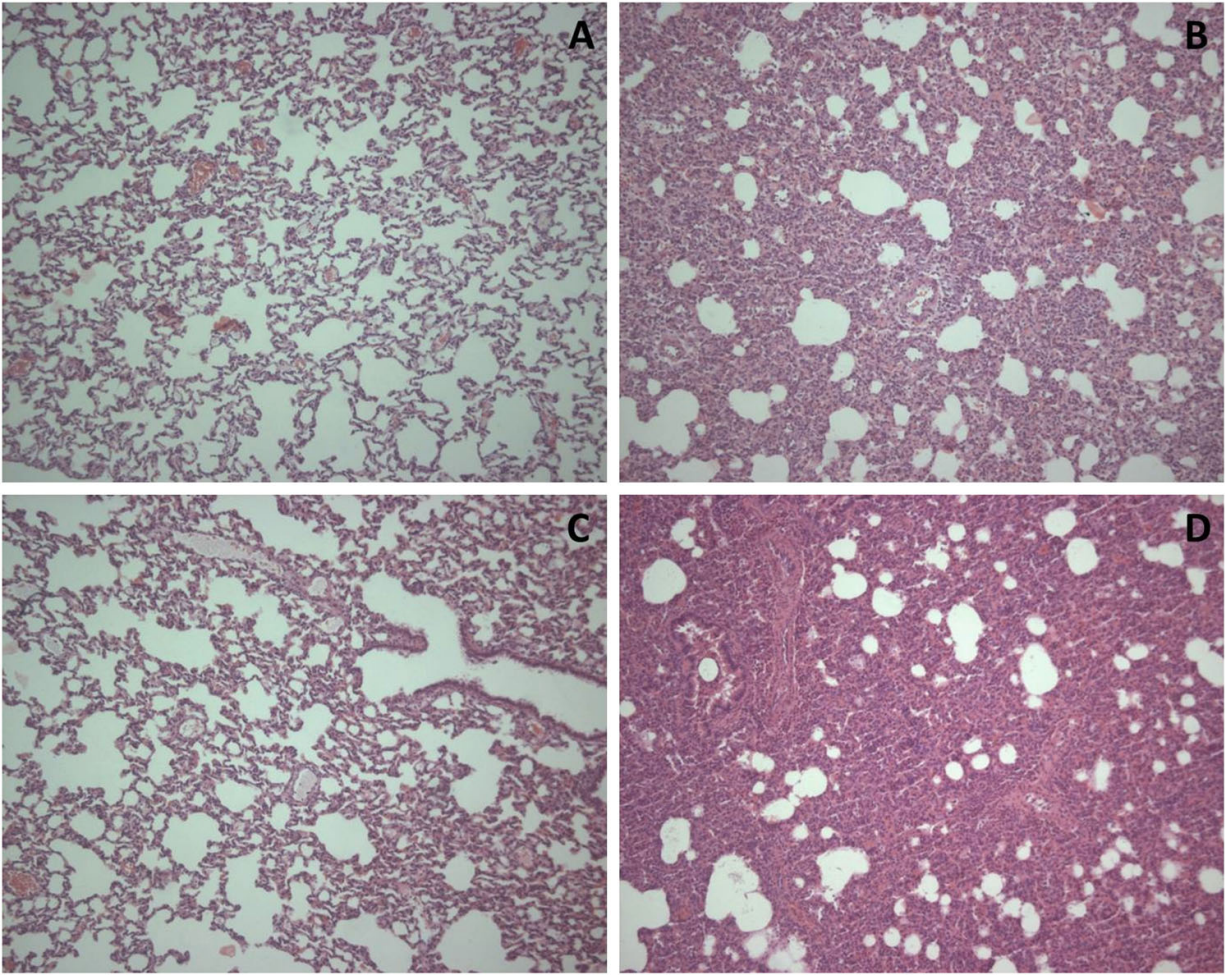
Haematoxylin and eosin stained lung sections of rats nebulized with saline 20 h after inoculation with sterile saline **a**; or *Streptococcus pneumoniae* **b**; and 40 h after inoculation with sterile saline **c**; or *Streptococcus pneumoniae* **d**

**Fig. 3:**
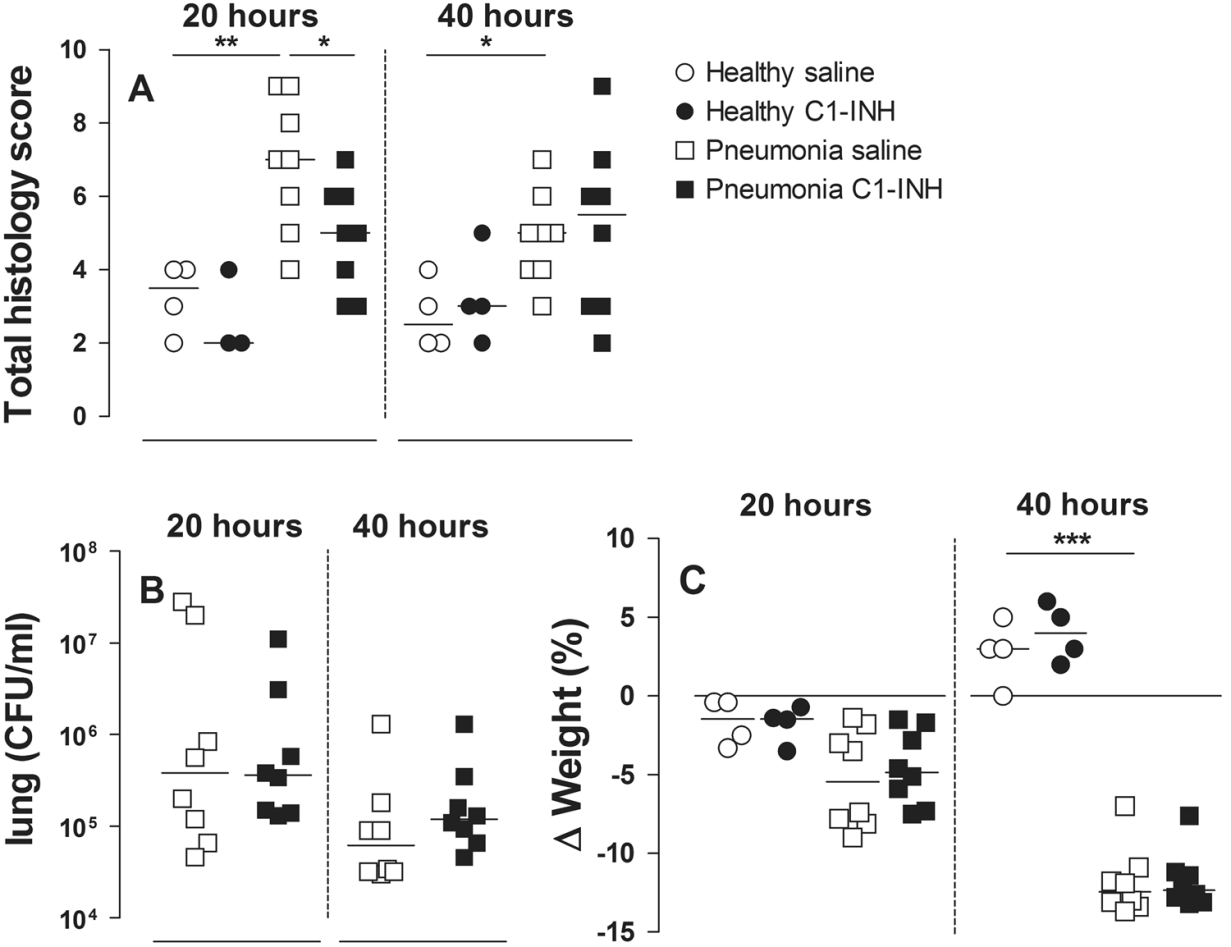
Total histopathology score **a** bacterial outgrowth in the lung **b** and change in bodyweight **c** in rats after 20 or 40 h in healthy animals (*circles*) or animals with *Streptococcus pneumoniae* pneumonia (*squares*). Animals were treated with either nebulized sterile saline (*clear*) or nebulized C1–INH (*black*). Bars represent median. *p < 0.05; **p < 0.01, and ***p < 0.001

All animals survived the experiment until the moment of sacrifice (Fig. 1). After inoculation with *S. pneumoniae,* rats developed severe pneumonia with dyspnea, and macroscopic infiltrates on post-mortem examination. Histopathologic analysis showed clear signs of pneumonia (Fig. 2) and total histology score was increased upon inoculation with *S. pneumoniae* after 20 and 40 h (Fig. 3a). Bacterial outgrowth of *S. pneumoniae* was observed in lung homogenates of infected rats (Fig. 3b) and the incidence of bacterial dissemination from the pulmonary compartment to the blood, increased from 12.5 % of the rats sacrificed 20 h after inoculation to 62.5 % of the rats sacrificed after 40 h. Twenty hours after inoculation with *S. pneumoniae* a non–significant decrease in bodyweight was observed. After 40 h this decrease in bodyweight was more pronounced and significant compared to healthy rats (Fig. 3c).

### C1-INH Treatment and Pulmonary Complement Activation

**Fig. 4:**
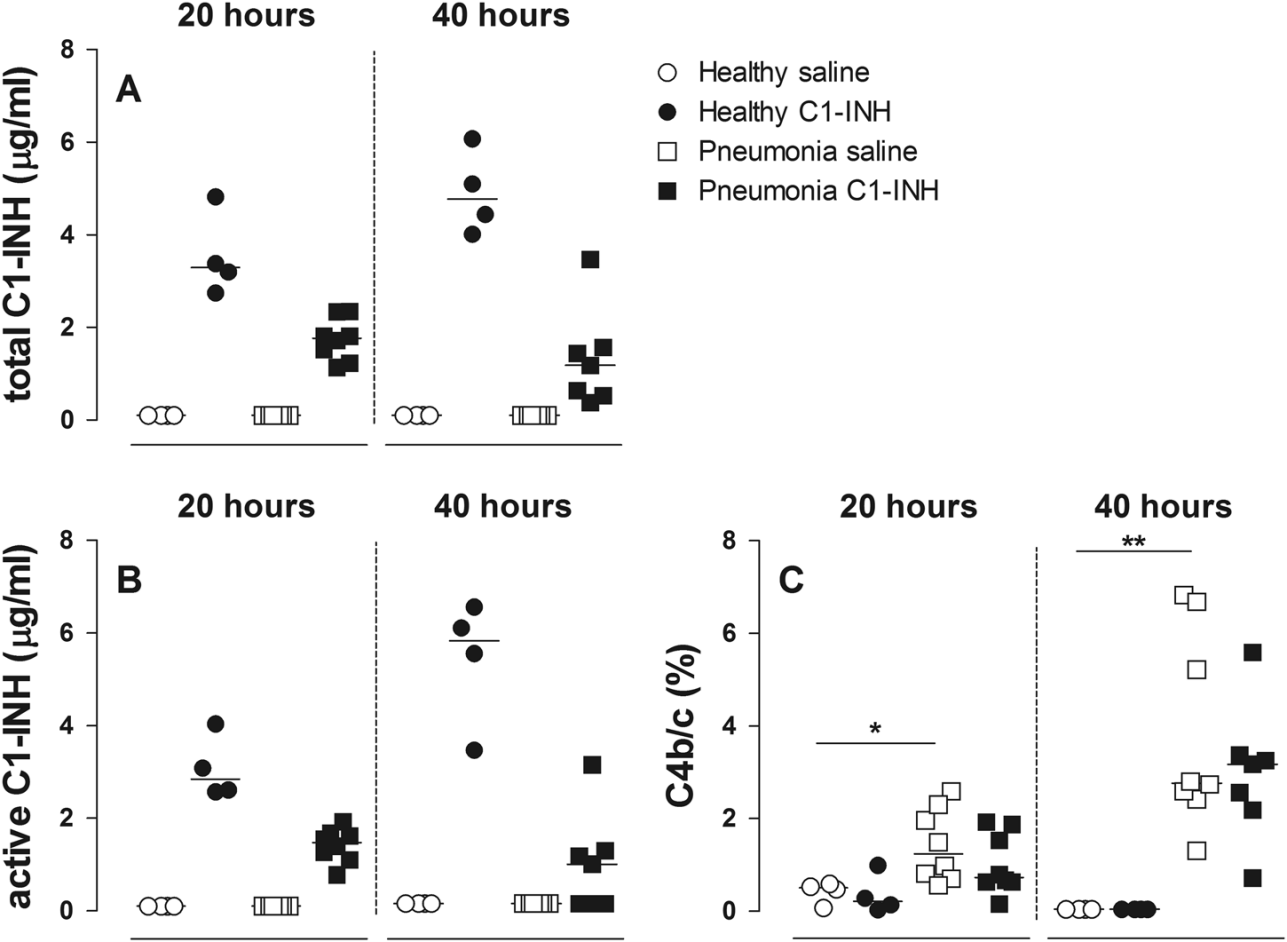
Total amount of human C1-INH antigen in BALF. **a** functional human C1-INH in BALF **b** C4b/c in BALF as a percentage of maximal activated C4b/c in plasma **c** in rats after 20 or 40 h in healthy animals (*circles*) or animals with *Streptococcus pneumoniae* pneumonia (*squares*). Animals were treated with either nebulized sterile saline (*clear*) or nebulized C1-INH (*black*). Bars represent median. *p < 0.05; **p < 0.01, and ***p < 0.001

In rats treated with nebulized C1-INH, significantly increased C1-INH levels (antigen and activity) were measured in BALF, compared to saline controls. This observation was found both at 20 and 40 h (Figs. 4a, b). Pneumonia resulted in augmented levels of C4b/c in BALF increasing over time. Despite the local C1-INH availability (including its active fraction), pulmonary C4b/c levels were not significantly attenuated at 20 and 40 h (Fig. 4c).

### Lung Inflammation and Injury

**Fig. 5:**
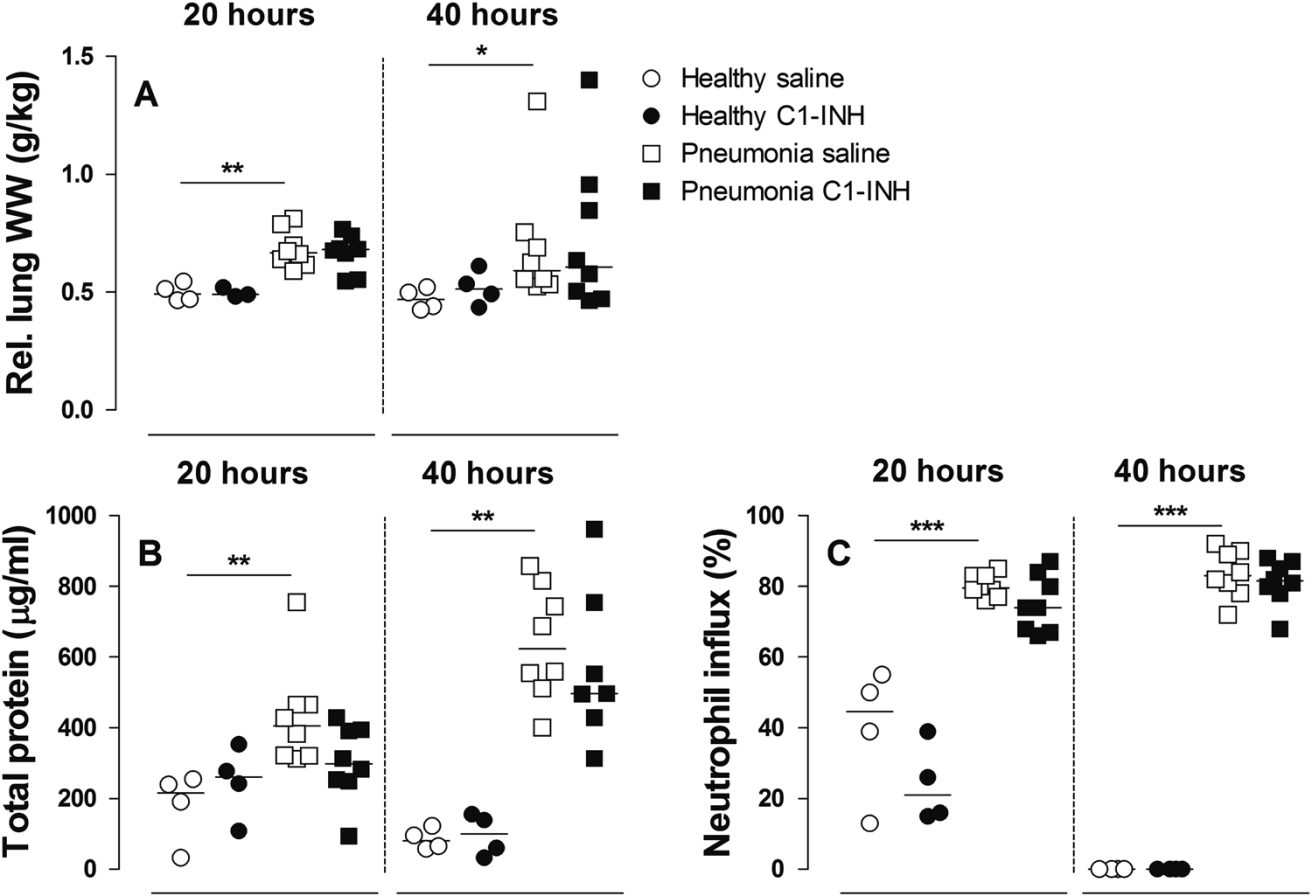
Relative lung wet weight. **a** total protein BALF **b** and neutrophil influx in BALF **c** in rats after 20 or 40 h in healthy animals (*circles*) or animals with *Streptococcus pneumoniae* pneumonia (*squares*). Animals were treated with either nebulized sterile saline (*clear*) or nebulized C1-INH (*black*). Bars represent median. *p < 0.05; **p < 0.01, and ***p < 0.001

C1-INH treatment showed a significant reduction of the total histology score at 20 h. This reduction was abrogated after 40 h of pneumonia (Fig. 3a). Relative lung wet weight (Fig. 5a), total protein (Fig. 5b), and neutrophil influx (Fig. 5c) were significantly increased at both 20 and 40 h. No effect of C1-INH treatment was seen on these lung injury parameters. In addition, bacterial outgrowth remained unchanged in C1-INH treated rats with pneumonia compared to their saline controls (12.5 % at 20 h and 62.5 % at 40 h).

**Fig. 6:**
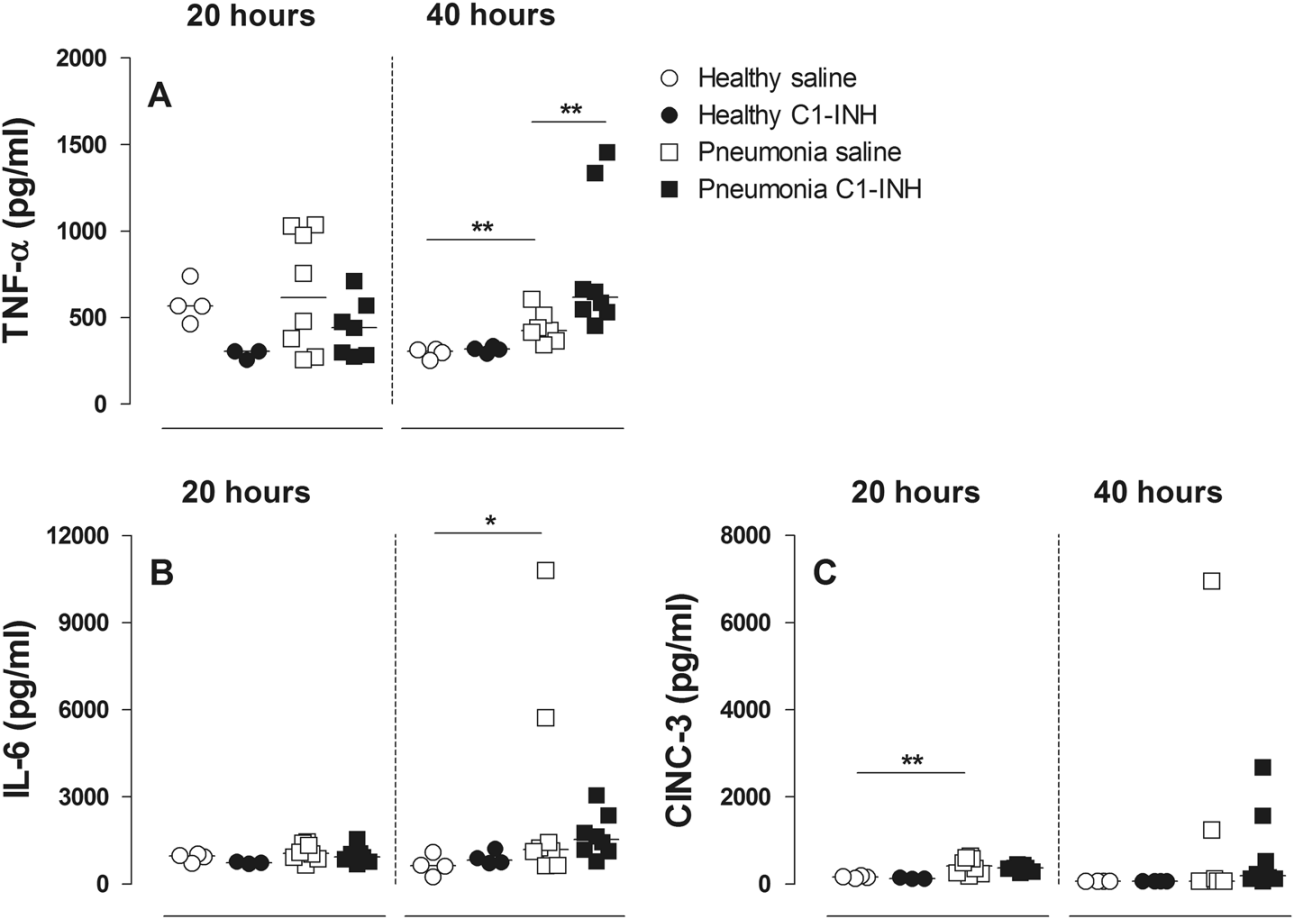
TNF-α **a** IL-6 **b** and CINC-3 **c** in BALF in rats after 20 or 40 h in healthy animals (*circles*) or animals with *Streptococcus pneumoniae* pneumonia (*squares*). Animals were treated with either nebulized sterile saline (*clear*) or nebulized C1-INH (*black*). Bars represent median. *p < 0.05; **p < 0.01, and ***p < 0.001

TNF-α and IL-6 were significantly increased in rats sacrificed after 40 h, but not after 20 h (Figs. 6a, b, respectively). In contrast, CINC-3 levels were significant increased at 20 h, though no differences were present at 40 h (Fig. 6c). Nebulized C1-INH did not reduce inflammatory markers IL-6 and CINC-3 (Figs. 6b, c, respectively), though higher levels of TNF-α were observed 40 h after inoculation in the C1-INH treated group (Fig. 6a).

## Discussion

In this experiment we demonstrate that severe pneumonia in rats is associated with complement activation in the lungs, measured by increased levels of complement C4b/c. After repetitive nebulization of C1-INH, both antigen and activity levels of C1-INH were detectable in the lungs. Despite these increased levels of C1-INH in the lungs, no effects on pulmonary complement activation (C4b/c), lung inflammation or lung injury were found.

In lung injury, local inflammatory reactions occur that comprises a complicated interaction between pneumocytes, inflammatory cells, cytokines, acute phase proteins, and the complement system [24]. In different animal models, complement activation was shown to result in tissue damage and organ dysfunction, including lung injury. Inhibition of complement activation was found to prevent these deleterious effects [12–18]. In this study, with rats we examined the extent and consequences of complement activation in the lungs during severe pneumonia.

So far, only two studies investigated inhibition of local complement activation in models of lung injury [19, 20]. Treatment with C1-INH reduced complement C3a levels, but not C5a levels in BALF in a rat model of transfusion-related acute lung injury [20]. A beneficial effect was found on the total histology score, but no effect was seen on vascular leakage or inflammation in the lung. In another study, in a two-hit model of pneumonia and injurious ventilation in rats, systemic treatment with C1-INH did not affect pulmonary complement activation, lung inflammation or lung injury [19]. Notably, in these two studies, C1-INH was administered systemically, which could have resulted in too low local levels of C1-INH. We chose to administer C1-INH locally by repetitive nebulization in our rat model of severe pneumonia. Nevertheless, we also found no beneficial effects, except for a short-lasting effect on the total histology score.

The lack of effect of nebulized C1-INH in our experiment may have several explanations. Aggravation of the pneumonia during the course of the disease might have overwhelmed the effects of administered C1-INH. Indeed, a beneficial effect was seen on the total histology score after 20 h, which is, at least in part in line with the effects observed in a previous study [20]. In our study this beneficial effect was no longer present after 40 h. Another explanation might be that complement activation is a first line of defense in fighting encapsulated bacteria and other humoral (e.g., antibodies and cytokines) and cellular immune responses eventually take over, contributing to increased tissue damage. However, no conclusions to that point can be drawn from these results, since this reduction in total histology score was not accompanied by an effect on pulmonary complement activation or on other endpoints.

It is also possible that nebulized C1-INH does not reach the smaller airways and alveoli of infected lungs. The rats with pneumonia had lower levels of C1-INH antigen and its active fraction in BALF compared to their healthy counterparts. This may have been caused by high frequent but low tidal breathing due to the pneumonia, scavenging of C1-INH by mucus in the airways or increased consumption of C1-INH due to the heightened inflammatory state and increased bacterial activity. Furthermore, increased proteolytic activity by elastase in the lung, catalyzed by an increased neutrophil influx, could cleave, and thus inactivate, the C1-INH protein. Such an increased proteolytic activity could also result in pulmonary complement activation further down the cascade [25]. In this situation, C1-INH, which regulates complement activation at the top of the cascade, will not be effective. Therefore, future experiments directed at pulmonary complement inhibition in lung injury models should consider different targets of intervention in the complement cascade (e.g. anti-C5 antibodies).

The nebulizer used for administration of drugs in this experiments has been applied in several previous rat lung injury studies in our laboratory. In these studies the effects of nebulized anti-coagulant and pro-fibrinolytic agents on pulmonary coagulopathy were investigated. Significant reduction in coagulation and increased fibrinolysis was described [21, 26]. This, at least, indicates good distribution of the aerosolized agents by the nebulizing technique used. In the present experiment we found C1-INH in BALF, though levels were below normal levels of C1-INH found in plasma. Therefore, one could state that underdosage may be another explanation for the absence of pulmonary complement inhibition. However, our C1-INH measurements are not corrected for dilution caused by the lung lavage. The dilution therefore largely underestimates the real concentration of C1-INH in the lung.

The increase of CINC-3 after 20 h of pneumonia and increased levels of TNF-α and IL-6 after 40 h of pneumonia probably reflects the inflammatory reactions during the *S. pneumoniae* pneumonia. However, the reason why TNF-α levels increase after 40 h of C1-INH treatment compared to saline controls remains unclear. It may reflect toxic effects of C1-INH, as has previously described by Müller et al. [20]. Previous studies showed C1-INH in high dosages to be able to induce inflammation rather than attenuate it [20, 27]. Since no increase in lung inflammation was observed in C1-INH treated groups in our study, with high levels of C1-INH in BALF, toxicity of C1-INH in this experiment seems unlikely.

The timing of C1-INH administration may be disputed. In our model, C1-INH was administrated before induction of pneumonia (pre-treatment), which is not the case in real life. Furthermore, antibiotics, as part of treatment following the guidelines, were not used during the experiment. For these reasons this study may be less clinically relevant. However, as C1-INH was ineffective in this model, it is not very likely that C1-INH administration later in the course of pneumonia would have revealed different results.

Although we were unable to reject our hypothesis, and taken into account the limitations of the study as described above, we consider our findings important for publication. Publication of negative animal studies is known to be very important in reducing publication bias in medical-scientific literature [28].

In conclusion, this is a negative experimental animal study in which repetitive nebulization with C1-INH, a potent inhibitor of the complement system, was examined. C1-INH was unable to attenuate pneumonia-induced pulmonary complement activation, lung inflammation and lung injury in severe *S. pneumonia* pneumonia in rats.
